# Breaking the bilayer: OMV formation during environmental transitions

**DOI:** 10.15698/mic2017.02.558

**Published:** 2017-02-03

**Authors:** Katherine E. Bonnington, Meta J. Kuehn

**Affiliations:** 1 Department of Biochemistry, Duke University Medical Center, Durham, North Carolina, USA.

**Keywords:** lipid A, outer membrane vesicles, Salmonella

## Abstract

Gram-negative bacteria maintain the barrier properties of the outer membrane (OM) in a wide array of physiological conditions despite their inability to degrade lipopolysaccharide (LPS) and protein material present in the outer leaflet of the OM. Through characterization of the native dynamics of outer membrane LPS change we recently described a mechanism in which these diderm organisms overcome this design flaw. In response to different environmental stimuli *Salmonella* *enterica* modulates the export of specific structural variants of lipid A via outer membrane vesicles (OMVs). We proposed that the polymorphic model for regulation of membrane lipid content could largely account for the structural differences between secreted and retained lipid A species. However, differences in OMV production levels and size observed between environmental conditions remain unexplained. Further exploration into the relationship between OMV production level and content specificity may shed light onto the enigmatic mechanisms of OMV formation.

The lipopolysaccharide content of the asymmetric Gram-negative outer membrane is highly heterogeneous. Each molecule may differ in the length of its O-antigen chain, modifications to its core region, covalent additions to the lipid A head group and/or differential acylation of the lipid A anchor. Proper balance of the bulk properties of this mixture of LPS molecules allows the membrane to retain its barrier functionality.

During logarithmic growth in the neutral pH (7.6H, pH 7.6 10 mM MgSO_4_) condition, we observed that the lipid A composition of the secreted OMVs directly paralleled the OM composition over time. The neutral pH and high levels of magnesium cations in the 7.6H-to-7.6H environment allow for the 1-diphosphate and 1,4’-bisphosphate hexacylated lipid A species to create a highly-crosslinked stable membrane structure. The OMVs produced in this condition were smaller and more protein-dense on average than OMVs produced in acidic media.

Two-component systems PmrA/B and PhoP/Q upregulate specific envelope modifications upon sensing acidic pH, toxic metals, cationic antimicrobial peptides, and low divalent cation concentrations. Consequently, lipid A structures modified with phosphoethanolamine (pEtN) and aminoarabinose (L-Ara4N) at the 1 and/or 4’ phosphates were added to the OM during cell growth in acidic media (5.8L, pH 5.8 10 µM MgSO_4_). These modifications neutralize the negatively charged phosphates which flank the lipid A head group, allowing the molecules to compensate for the loss of the divalent cation bridges. Additionally, the PhoP/Q-activated OM enzyme PagP was activated in the 5.8L conditions, resulting in palmitoylation of a variety of lipid A species in order to maintain the membrane barrier. In general, covalent modification of the lipid A head groups with pEtN and L-Ara4N decreased the likelihood of that molecule’s incorporation into OMVs. In contrast, hepta-acylated species created through palmitoylation were more likely to be found in secreted OMVs. Concomitantly, OMV production levels and size increased in the 7.6H-to-5.8L, 5.8L-to-5.8L, and 5.8L-to-7.6H conditions.

These observations are in agreement with the well-established model of polymorphic regulation of lipid composition. In this model, a regulated balance between bilayer-promoting and non-bilayer-promoting lipids is maintained throughout different environmental conditions. The geometry of each lipid molecule contributes to its intrinsic preference for lamellar or non-lamellar phases. Lipids with a more conical shape prefer non-lamellar phases (hexagonal, H_II_) and aid in the transition to nonbilayer structures, as would occur during OMV formation. As increased preference for the H_II _phase occurs when hydrocarbon chain length or splay increases and decreases with effective head group size increase, the higher levels of palmitoylated lipid A species and lower levels of covalently modified species we observe in OMVs produced in 5.8L conditions fit well with the polymorphic regulation model (Figure 1).

**Figure 1:**
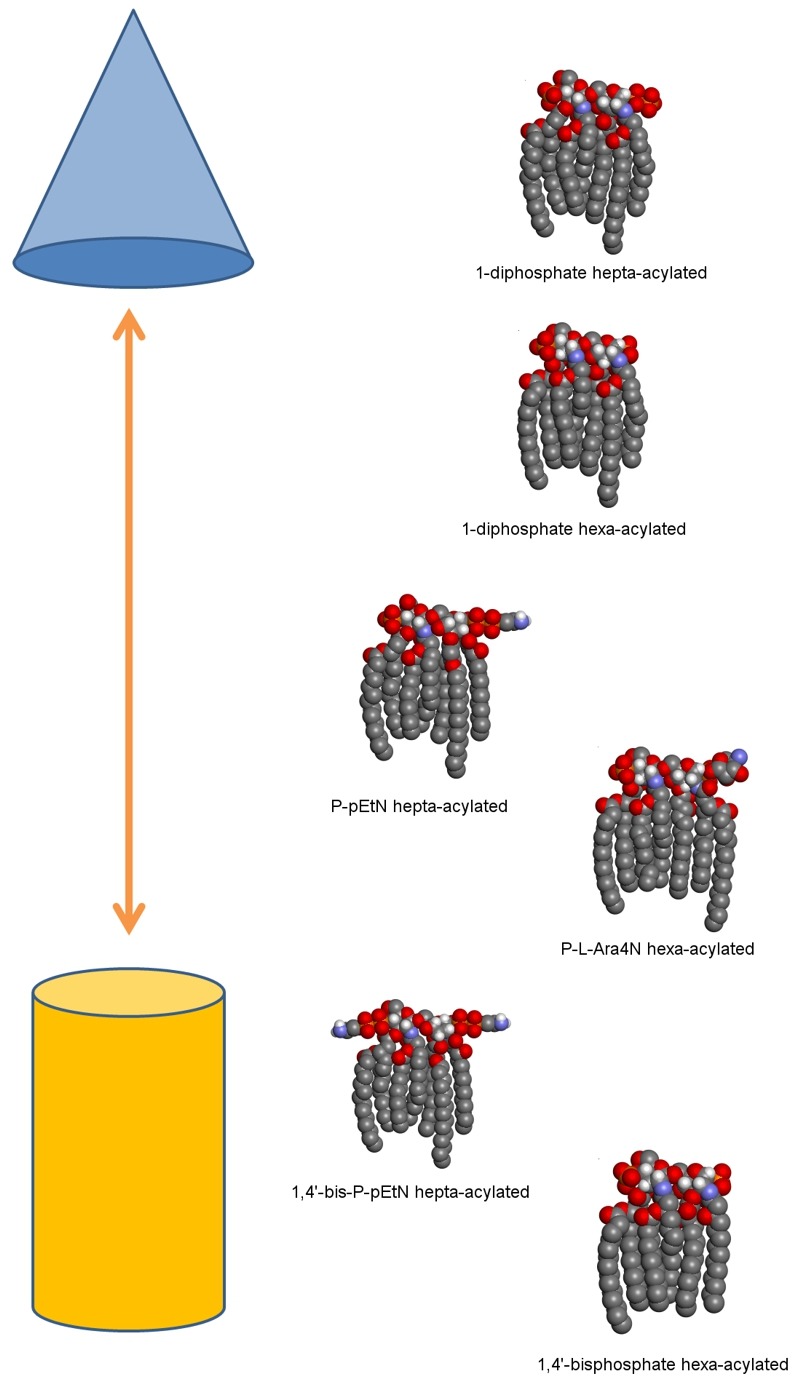
FIGURE 1: Model illustrating the diversity of lipid A structures and their preference for non-lamellar phase to lamellar phase in acidic conditions. Lipid A molecules are shown as three-dimensional space-filling models in typical Corey-Pauling-Koltun coloring (with the majority of hydrogen atoms omitted for simplicity). The proposed relative propensity of each lipid A structure towards the hexagonal phase (top, represented by a blue cone) or towards the lamellar phase (bottom, represented by an orange cylinder) is shown here. From top to bottom these lipid A structures are shown from most to least likely to be found secreted in OMVs during 5.8L conditions according to ESI-MS data.

To probe the relationship of non-lamellar-promoting lipids with OMV production levels, we quantified the OMV production levels of *phoP^c^* (a mutant in which *phoP* is constitutively active) and *phoP^c^pagP-* (*pagP* deletion in the *phoP^c^*background) strains (generously provided by SI Miller). We find that the constitutive expression of the *phoP* operon results in higher levels of OMV production in both the 7.6H and 5.8L conditions (Figure 2). Deletion of *pagP* in the *phoP^c^* background lowered this production back to near WT levels in both environmental conditions. As the PagP-catalyzed palmitoylation of lipid A may serve to stabilize non-lamellar phases, its responsibility for higher production levels in the *phoP^c^* strain adds further support for the polymorphic regulation model.

**Figure 2:**
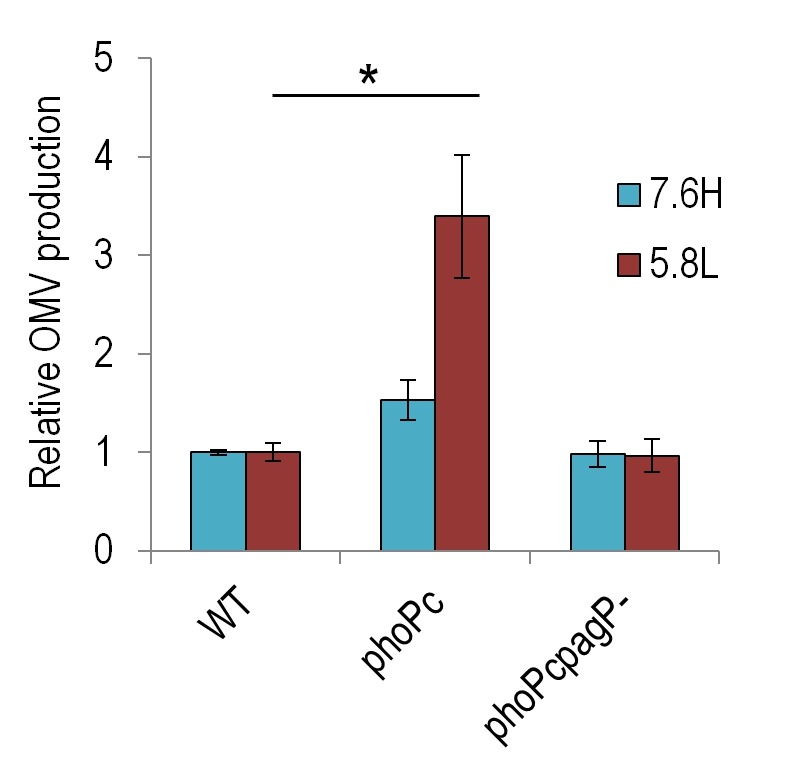
FIGURE 2: Constitutive activation of *phoP* increases OMV production in 7.6H and 5.8L conditions. The relative OMV production, normalized to CFU, during overnight growth of WT (14028s), *phoP^c^* (14028s *pho-24*), and *phoP^c^pagP-* (14028s *pho-24 pagP::TnPhoA*) strains in 7.6H (N-minimal media pH 7.6 10 mM MgSO_4_) and 5.8L media (N-minimal media pH 5.8 10 µM MgSO_4_), as calculated for protein content by OMP densitometry. For all conditions, n > 3; *, statistically significant difference for the indicated pair, p < 0.005.

Furthermore, changes to the OM lipid environment, in both leaflets, may influence the conformation of OMPs and the ease of which proteins fold or fit into the membrane. As cells prefer different suites of OMPs in different environmental conditions, environmentally-triggered changes to the permeability of an OMP could favor its removal from the OM. In order to incentivize this result, bacterial cells could build a fail-safe into their system by relying on lipid changes to execute the necessary protein removal. If shifting lipid content results in non-ideal folding conditions for certain OMPs, OMVs could provide a convenient means of disposal for these unfolded proteins. Additionally, if the new lipid environment results in a mismatch between the hydrophobic regions of proteins and the hydrophobic membrane space, then strain from the membrane bending in order to accommodate the inappropriate protein will tip the scales towards membrane bending and vesicle formation.

We found that extended exposure to mildly acidic, low-magnesium conditions correlated with increased average diameter of secreted OMVs. One membrane feature which may influence OMV size may be the steric factors relating to lipid A head group size and propensity for intermolecular crosslinking. If differently modified structures contribute differently to the membrane bending modulus, then these intrinsic structural properties could lead to a preferred OMV size distribution per characteristic lipid mix.

We also considered that reduction of the level of OM-peptidoglycan (PG) connectivity might lead cells to shed larger vesicles. Although we did not find significant differences in the levels of the lipoprotein Lpp covalently attached to PG between the 7.6H-to-7.6H, 7.6H-to-5.8L, and 5.8L-to-5.8L conditions, changes in other mechanisms of attachment between the OM and PG layer may still occur or develop after further exposure to acidic conditions. Environmentally-regulated alterations to peptidoglycan metabolism and changes in OMP composition, especially in OmpA, could affect the level of OM-PG crosslinking.

In the phospholipid membranes, presence of nonbilayer lipids can influence peripheral and transmembrane protein function. Accordingly, the increased levels of OM cardiolipin observed under PhoP/Q-activating conditions could play additional role in OMV formation through the modulation of protein functionality. The activation or inhibition of protein activities in the inner leaflet of the OM could affect the membrane’s connectivity to the PG layer, remodeling of the PG sacculus, and other periplasmic processes.

OMV production levels could be influenced by these same factors, along with the afore-mentioned degree of OM attachment to the PG layer. Effects of the environment on periplasmic-localized processes could also influence OMV production levels in as-of-yet unknown ways.

Content selection in OMV formation is likely principally influenced by innate bilayer-forming propensity of areas of membrane. Therefore the processes involved in OM biogenesis would play a major role in dictating the potential of certain areas of membrane to bleb. Content selection could also be influenced by the interactions between individual lipid molecules with each other and the lateral surface of OMPs. However, since we do not observe any large differences between the protein composition of the OM and OMV during any of our environmental shifts utilizing SDS-PAGE, we cannot yet discern if these smaller intermolecular interactions to play a significant role in content selection.

While many questions about OMV formation and the relationship between production levels and content selection remain, further probing into well-characterized systems may yield insights in the future. Additionally, the disparity in LPS composition between the two membrane structures could have interesting implications for the processes of host immune activation and pathogen immune evasion.

